# Efficacy of adjuvant metronidazole therapy on peri-implantitis: a systematic review and meta-analysis of randomized clinical studies

**DOI:** 10.3389/fcimb.2023.1149055

**Published:** 2023-05-23

**Authors:** Nansi López-Valverde, Antonio López-Valverde, José Antonio Blanco-Rueda

**Affiliations:** ^1^ Department of Medicine and Medical Specialties, Faculty of Health Sciences, Universidad Alcalá de Henares, Madrid, Spain; ^2^ Department of Surgery, Instituto de Investigación Biomédica de Salamanca (IBSAL), University of Salamanca, Salamanca, Spain

**Keywords:** dental implants, peri-implantitis, antibacterial agents/therapeutic use, metronidazole, RCTs, meta-analysis

## Abstract

Peri-implant diseases are pathological conditions that affect the survival of dental implants. Etiological studies are limited, accepting a prevalence of 20% at the implant level and 24% at the patient level. The benefits of adjuvant metronidazole are controversial. A systematic review and meta-analysis of RCTs according to PRISMA and PICOS was performed with an electronic search over the last 10 years in MEDLINE (PubMed), WOS, Embase, and Cochrane Library. The risk of bias was measured using the Cochrane Risk of Bias tool and the methodological quality using the Jadad scale. Meta-analysis was performed with RevMan version 5.4.1, based on mean difference and standard deviation, with 95% confidence intervals; the random-effects model was selected, and the threshold for statistical significance was defined as *p* < 0.05. A total of 38 studies were collected and five were selected. Finally, one of the studies was eliminated because of unanalyzable results. All studies reached a high methodological quality. A total of 289 patients were studied with follow-up periods from 2 weeks to 1 year. Statistical significance was only found, with respect to the use of adjunctive metronidazole, in the pooled analysis of the studies (*p* = 0.02) and in the analysis of the radiographic values reported on peri-implant marginal bone levels, in the studies with a 3-month follow-up (*p* = 0.03). Discrepancies in the use of systemic metronidazole require long-term randomized clinical trials (RCTs) to determine the role of antibiotics in the treatment of peri-implantitis.

## 1 Introduction

Mucositis and peri-implantitis are considered peri-implant diseases associated with biofilms and affect osseointegrated implants (Berglundh et al., 2018). For clinicians to establish a clear differentiation with the peri-implant health status and apply a correct treatment, these pathologies require a clear definition, and it is generally accepted that mucositis is a reversible inflammation of the peri-implant soft tissues, and peri-implantitis, induced by plaque, is considered one of the most frequent late biological complications in dental implantology, affecting hard and soft tissues, generating a loss of peri-implant bone support, and reducing osseointegration (Schwarz et al., 2006; Smeets et al., 2014). It is usually associated with bleeding in the peri-implant sulcus, spontaneous or provoked, increased suppuration, and increased probing depth of the peri-implant pocket (Schwarz et al., 2018).

Dental implantology has made it necessary to investigate the oral microbiota in certain pathogenic situations, with the aim of demonstrating that the formation of biofilms is a determining factor in the loss of peri-implant bone support. However, despite the similarity with periodontal diseases, etiological studies of peri-implant diseases are limited, and common causative organisms have been identified for both pathologies, mainly gram-negative anaerobic bacteria, such as *Porphyromonas gingivalis*, *Prevotella intermedia*, *Tannerella forsythia*, *Eikenella corrodens*, *Filifactor alocis*, *Aggregatibacter actinomycetemcomitans*, and *Staphylococcus aureus*; however, there is controversy about the homogeneity of biofilm composition in both pathologies (Persson and Renvert, 2014; Sahrmann et al., 2020; Jiang et al., 2021; Mulla et al., 2021).

Precisely, the different diagnostic definitions condition the ranges of their prevalence, lower in peri-implantitis than in mucositis, with an approximate range, for the latter, between 28% and 77% of the subjects and between 12% and 43% of the implant sites (Zitzmann and Berglundh, 2008; Koldsland et al., 2010). A recent study on 474 implants in 275 patients showed peri-implantitis with a prevalence of 20% at the implant level and 24% at the patient level (Zitzmann and Berglundh, 2008).

Surgical and non-surgical treatments of peri-implantitis, based on the scientific evidence of periodontal treatments, are aimed at controlling infection and reducing the bacterial load, and it is known that surgical treatments alone have proven to be ineffective (Renvert et al., 2008; Rodrigo et al., 2018). The benefits of local or systemic antibacterials, used as adjuvants, have also been demonstrated (Liñares et al., 2019), and it is a frequent practice to prescribe systemic antibiotics for the treatment of peri-implant diseases and other dental pathologies, since, in addition to exerting an antimicrobial effect, they facilitate healing (Javed et al., 2013).

In peri-implant lesions, it has been observed that non-surgical treatments combined with systemic metronidazole reduce the probing depth (Liñares et al., 2019), although some research has indicated that the efficacy of adjuvant antibiotics in the non-surgical treatment of peri-implantitis may be conditioned by the severity of the disease (van Winkelhoff, 2012; Park et al., 2021).

Therefore, this systematic review and meta-analysis aimed to address the following specific question: In patients with peri-implantitis, is adjuvant local or systemic treatment with metronidazole effective on signs of inflammation and bone destruction?

## 2 Materials and methods

The review protocol was developed and structured according to the Preferred Reporting Items for Systematic Review and Meta-Analyses (PRISMA) statement (Page et al., 2021) (**Table 1**). The review was registered in INPLASY under registration number INPLASY202310015.

**Table 1:** PRISMA checklist.

Section/topic	#	Checklist item	Reported on page #
TITLE: Efficacy of metronidazole on peri-implantitis: a systematic review and meta-analysis of randomized studies.			
Title	1	Systematic review and meta-analysis.	1
ABSTRACT			
Structured summary	2	Peri-implant diseases are pathological conditions that affect the survival of dental implants. Etiological studies are limited, accepting a prevalence of 20% at the implant level and 24% at the patient level. The benefits of adjuvant metronidazole are controversial. A systematic review and meta-analysis of RCTs according to PRISMA and PICOS was performed with an electronic search over the last 10 years in MEDLINE (PubMed), WOS, Embase, and Cochrane Library. The risk of bias was measured using the Cochrane Risk of Bias tool and methodological quality using the Jadad scale. Meta-analysis was performed with RevMan version 5.4.1, based on mean difference and standard deviation, with 95% confidence intervals; the random-effects model was selected; the threshold for statistical significance was defined as *p* < 0.05. A total of 38 studies were collected and five were selected. Finally, one of the studies was eliminated because of unanalyzable results. All studies achieved high methodological quality. A total of 289 patients were studied with follow-up periods from 2 weeks to 1 year. Statistical significance was found in the pooled analysis of the studies (*p* = 0.02). Discrepancies in the use of systemic metronidazole call for long-term RCTs to determine the role of antibiotics in the treatment of peri-implantitis. INPLASY202310015	1
INTRODUCTION			
Rationale	3	Peri-implantitis is a group of pathologies with a prevalence of 20%, which causes a good number of dental implant losses, resulting in economic and social disruption. Adjuvant antibiotic treatments, local or systemic, can be used to treat peri-implantitis, together with non-surgical treatments, with the main objective of controlling the infection and reducing the bacterial load. Our study presents a systematic review and meta-analysis of RCTs, according to PRISMA, with the aim of determining the efficacy of adjuvant metronidazole treatment, local or systemic, on inflammation and bone destruction. Given the existing discrepancies in the use of local or systemic metronidazole, alone or in combination with other antibiotics, as adjuvant treatment in peri-implantitis, RCTs with prolonged follow-up are recommended to determine the exact role of antibiotics in the treatment of peri-implantitis.	1
Objectives	4	This systematic review and meta-analysis aimed to address the following specific question: In patients with peri-implantitis, is adjuvant local or systemic treatment with metronidazole effective on signs of inflammation and bone destruction?	2
METHODS			
Protocol and registration	5	INPLASY202310015 Doi: 10.37766inplasy2023.1.0015	2
Eligibility criteria	6	PICOS Inclusion criteria: a) RCTs (single or double-blind) performed in patients with peri-implantitis defined as bleeding bone loss ≥2 mm and/or suppuration on peri-implant probing (≥4 mm) b) Studies comparing the efficacy of local/systemic metronidazole adjuvant therapy *vs*. single surgical or non-surgical treatment c) Articles published in English	2, 3
Information sources	7	PubMed/MEDLINE; WOS; EMBASE; Cochrane Library	3
Search	8	Present a full electronic search strategy for at least one database, including any limits used, such that it could be repeated.	3
Study selection	9	RCTs	3
Data collection process	10	The titles and abstracts of the selected articles were collected and entered into an Excel spreadsheet, eliminating studies that did not refer to the research question.	
Data items	11	PICOS	3
Risk of bias in individual studies	12	Two reviewers (NL-V and AL-V) independently assessed the quality of each RCT according to the Cochrane Risk of Bias tool (RoB2).	3
Summary measures	13	Difference in means.	4
Synthesis of results	14	*I* ^2^ for each meta-analysis.	4
Risk of bias across studies	15	Funnel plot	4
Additional analyses	16	—————	
RESULTS			
Study selection	17	A total of 38 studies were collected, of which 11 were from MEDLINE-PubMed, 9 from WOS, and 9 from Embase, and 5 were finally selected for meta-analysis. We obtained a good inter-reviewer agreement, with a Cohen’s kappa index of *κ* = 80%.	3
Study characteristics	18	The studies with the largest sample size were a multicenter study on 118 patients and the study by De Waal et al. on 62 patients. The longest follow-up (12 months) was in the studies by Blanco et al. and De Waal et al. The study by Blanco et al. was the only one to use systemic metronidazole *vs.* placebo; the studies by Polymeri et al., De Waal et al., and Shibli et al. used amoxicillin together with metronidazole. The study by Park et al. used metronidazole and minocycline topically. Only the studies by Blanco et al. and Park et al. supported the use of metronidazole as an adjuvant in the treatment of peri-implantitis.	5–8
Risk of bias within studies	19	Of the five included studies, only four were included in the meta-analysis; the study by Polymeri et al. (2022) was excluded for presenting data in a non-analyzable form. All studies met the domains of random sequence generation and blinding of participants and personnel; one study was biased in the incomplete outcome domain and all had unclear data in the domains of incomplete outcome, blinding of outcome assessment, and allocation concealment. The study by Blanco et al. was the highest rated.	9
Results of individual studies	20	——————————	4
Synthesis of results	21	Heterogeneity was moderate in the analysis of grouped studies (*I* ^2 ^= 45%). In the analysis by subgroups, total homogeneity was found in subgroup 2 (*I* ^2 ^= 0%) (**Figure 1**); subgroups 1 and 3 obtained moderate heterogeneity (*I* ^2 ^= 46% and *I* ^2 ^= 37%, respectively). Statistical significance was found in the pooled study analysis (*p* = 0.02) (95% CI).	4
Risk of bias across studies	22	———————————	
Additional analysis	23	———————————	
DISCUSSION			
Summary of evidence	24	We suggest that the decision for a particular adjuvant antibiotic treatment should be made individually for each patient, considering the severity and extent of concomitant periodontitis, as well as the number of peri-implantitis areas affected and in need of treatment. Likewise, the potential risk of the development of strains resistant to amoxicillin and metronidazole should be taken into consideration and the indicated situations should be chosen carefully.	13
Limitations	25	There are limitations in our meta-analysis. On the one hand, the included studies used local and systemic adjuvant antibiotics; on the other hand, only the study by Blanco et al. used metronidazole as adjuvant treatment; all the others used metronidazole combined with other antibiotics.	15
Conclusions	26	There are discrepancies in the use of local or systemic metronidazole, alone or in combination with other antibiotics, as adjunctive treatment in peri-implantitis.	15
FUNDING			
Funding	27	No funding	

### 2.1 PICOS and focused question

The focused question used for the literature search was structured according to the Participants, Interventions, Control, Outcomes, Study design (PICOS) format: In patients with peri-implantitis, is adjuvant local or systemic treatment with metronidazole effective on changing signs of inflammation and bone destruction?

(P) Population: patients receiving adjuvant treatment with local or systemic metronidazole.
(I) Intervention: surgical or non-surgical treatment of peri-implantitis.
(C) Comparison: patients not receiving adjuvant treatment with metronidazole.
(O) Outcomes: primary outcomes: bleeding on probing, bleeding rate, suppuration, and probing depth. Secondary outcomes: clinical attachment level, bone level (radiographic test), and implant failure.
(S) Study design: randomized controlled trials.

### 2.2 Information source and search strategy

Four electronic databases were searched for relevant articles published in the last 10 years up to December 2022: MEDLINE (through PubMed), WOS, Embase, and Cochrane Library. The search filter “Randomized Controlled Trial” was applied. The electronic search was complemented with a manual search in the following journals: *Clinical Implant Dentistry and Related Research*, *Clinical Oral Implants Research*, *International Journal of Oral and Maxillofacial Implants*, *Journal of Clinical Periodontology*, and *Journal of Periodontology*.

The following Medical Subject Headings (MeSH) terms were used: “Dental implants,” “Dental plaque,” “Peri-Implantitis/prevention and control,” “Anti-Bacterial Agents/therapeutic use,” “Metronidazole/therapeutic use,” and “Humans.” Boolean AND/OR operators were used to refine the search. In addition, relevant studies from the grey literature and reference lists of the included studies (cross-references) were also examined. The search strategy and PICOS format are shown in **Table 2**.

**Table 2:** The search strategy and PICOS format.

Population	Patients receiving adjuvant treatment with local or systemic metronidazole
Intervention	Surgical or non-surgical treatment of peri-implantitis
Comparisons	Patients not receiving metronidazole as adjuvant treatment
Outcomes	Primary outcomes: bleeding on probing, bleeding rate, suppuration, and probing depth. Secondary outcomes: clinical attachment level, bone level (radiographic test), and implant failure
Study design	Randomized controlled trials (RCTs)
Search combination	#1 AND #2 OR
Language	English
Electronic databases	PubMed/MEDLINE; WOS; EMBASE; Cochrane Library

### 2.3 Inclusion and exclusion criteria

The inclusion criteria were as follows:

a) RCTs (single or double-blind) performed in patients with peri-implantitis defined as bleeding, bone loss ≥2 mm, and/or suppuration on peri-implant probing (≥ 4 mm)
b) Studies comparing the efficacy of local/systemic metronidazole adjuvant therapy *vs*. single surgical or non-surgical treatment
c) Articles published in English

The exclusion criteria were as follows:

a) Less than five patients per treatment group
b) Lack of clinical or radiographic data on bone destruction
c) Case series or clinical cases
d) Undefined cases and non-relevant studies

### 2.4 Data extraction and analysis and study selection

The titles and abstracts of the selected articles were collected and entered into an Excel spreadsheet, eliminating studies that did not refer to the research question. Two reviewers (NL-V and AL-V) independently selected titles and abstracts. Cohen’s kappa index (*κ*) (Cohen, 1968) was calculated to determine the degree of agreement between reviewers, and discrepancies between the two reviewers, regarding the inclusion of eligible studies, were reviewed and discussed by a third reviewer (JAB-R).

Subsequently, the selected articles were obtained for review, data extraction, and inclusion. Bibliographic references of the included studies were also reviewed as possible sources of additional studies.

### 2.5 Risk of bias of the included studies

Two reviewers (NL-V and AL-V) independently assessed the quality of each RCT according to the Cochrane Risk of Bias tool (RoB2) (Minozzi et al., 2022). Five domains of bias (randomization process, deviations from intended interventions, missing outcome data, outcome measurement, and selection of reported outcomes) were assessed. The Cochrane Handbook for Systematic Reviews of Interventions was used. The rating “high” indicated a high risk of bias, “low” indicated a low risk of bias, and “borderline” indicated the presence of bias due to uncertainty or lack of information about possible bias. Studies were classified as low, high risk of bias, or borderline. Any discrepancies in the assessment of RoB2 were discussed between the two reviewers with the aim of reaching a consensus.

**Figure 1:**
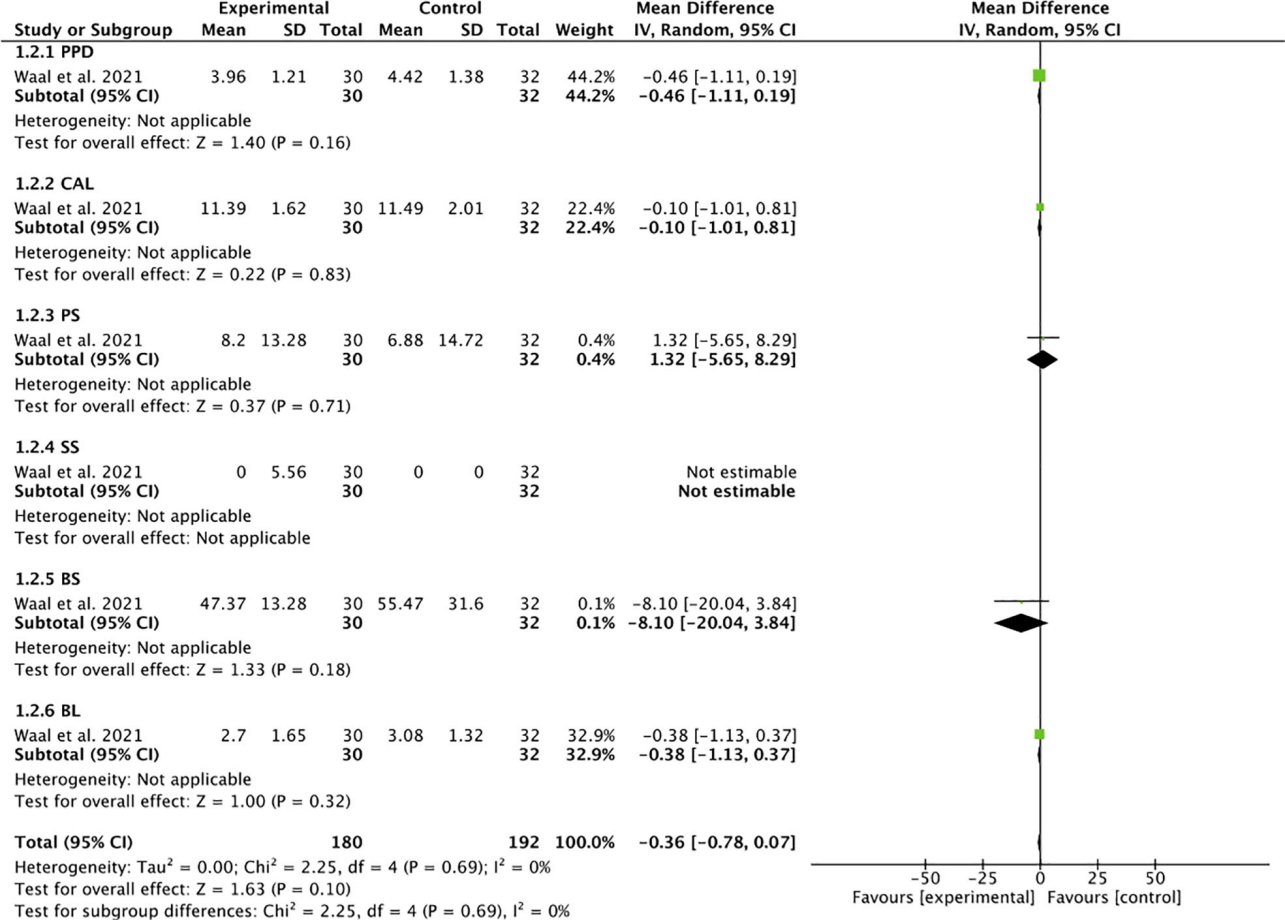
Forest plot for subgroup 2 (PPD, CAL, PS, SS, BS, BL).

### 2.6 Quality of the reports of the included studies

The Jadad scale (Oxford quality scoring system) (Jadad et al., 1996) defines the methodological quality of the studies based on the description of randomization, blinding, and dropouts, and was used to assess the methodological quality of the included studies. The scale ranges from 0 to 5: a score ≤2 means low quality of the reports and a score ≥3 means high quality.

### 2.7 Data analysis

Meta-analysis was performed using Review Manager (RevMan Software version 5.4.1; The Cochrane Collaboration, Copenhagen, Denmark; 2020). A meta-analysis was performed based on the mean difference (MD) and standard deviation (SD) to estimate effect size, with 95% confidence intervals (CI) for adverse event outcomes. The random-effects model was selected considering the uncertainty in *I* ^2^ when few studies are used in the meta-analysis due to the expected methodological heterogeneity in the included studies. The heterogeneity was interpreted as follows: low *I* ^2 ^= 25%, moderate *I* ^2 ^= 50%, and high *I* ^2 ^= 75%. The threshold for statistical significance was defined as *p* < 0.05.

## 3 Results

### 3.1 Characteristics of the studies

**Figure 2:**
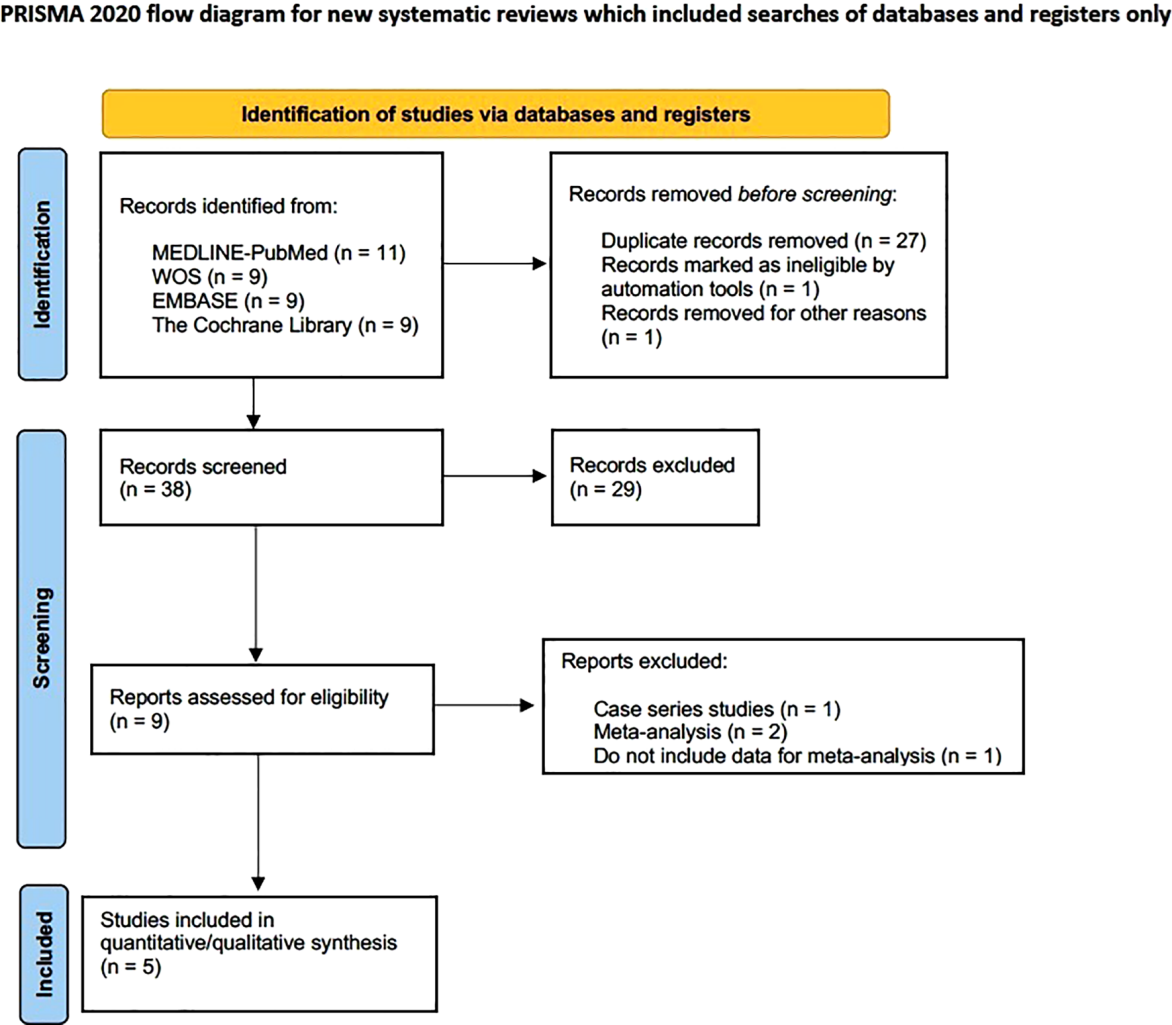
Flow diagram.

A total of 38 studies were collected, of which 11 were from MEDLINE-PubMed, 9 from WOS, 9 from Embase, and 9 from the Cochrane Library; finally, five (Shibli et al., 2019; De Waal et al., 2021; Park et al., 2021; Blanco et al., 2022; Polymeri et al., 2022) were finally selected for meta-analysis. A good inter-reviewer agreement was obtained with a Cohen’s kappa index of *κ* = 80% (**Figure 2**).

The studies with the largest sample size were a multicenter study on 118 patients (Park et al., 2021) and the study by De Waal et al. (2021) on 62 patients. The longest follow-up (12 months) was in the studies by Blanco et al. and De Waal et al (De Waal et al., 2021; Blanco et al., 2022). The study by Blanco et al. (2022) was the only one to use systemic metronidazole *vs*. placebo; the studies by Polymeri et al., De Waal et al., and Shibli et al (Shibli et al., 2019; De Waal et al., 2021; Polymeri et al., 2022). used amoxicillin together with metronidazole. The study by Park et al. (2021) used metronidazole and minocycline topically. Only the studies by Blanco et al. and Park et al (Park et al., 2021; Blanco et al., 2022). supported the use of metronidazole as an adjuvant in the treatment of peri-implantitis (**Table 3**).

**Table 3:** General characteristics of the selected studies.

Study; aim	Participants; groups	Interventions	Follow-up	Outcomes	Clinical parameters assessed
Blanco et al., 2022 Aim: To study the clinical and radiographic results after non-surgical treatment of peri-implantitis, with or without adjuvant systemic metronidazole (Blanco et al., 2022)	32 subjects; two groups (16 experimental and 16 control)	Implants received a mechanical non-surgical debridement session and systemic metronidazole or placebo	3, 6, and 12 months	The adjunctive use of systemic metronidazole as an adjunct to non-surgical treatment of peri-implantitis resulted in significant additional improvements in clinical and radiographic parameters after 12 months of follow-up	PPD, REC, CAL, BoP, PS, BS, RBL
Polymeri et al., 2022 Aim: To assess the adjunctive effect of systemic amoxicillin and metronidazole in patients receiving non-surgical treatment for peri-implantitis (Polymeri et al., 2022)	37 patients; two groups (18 experimental and 19 control)	After a session of mechanical debridement and treatment of the implant surfaces with ultrasound and hand instruments, the patients were treated with amoxicillin and metronidazole	12 weeks	The study found no clinical benefit with the adjunctive use of systemic amoxicillin and metronidazole in the non-surgical treatment of peri-implantitis	PIPD, BoP, SoP, PS
De Waal et al., 2021 Aim: To evaluate the complementary clinical and microbiological effect of systemic amoxicillin plus metronidazole in the non-surgical treatment of peri-implantitis (De Waal et al., 2021)	62 patients; two groups (30 experimental and 32 control)	Implants were supra- and submucosally cleaned using an air polisher with a subgingival tip and ultrasonic instruments. The test group patients additionally used systemic amoxicillin and metronidazole	12 weeks	Adjuvant systemic antibiotic treatment of amoxicillin and metronidazole does not improve the clinical and microbiological results of non-surgical treatment of peri-implantitis and should not be routinely recommended	PPD, CAL, PS, SoP, BS, BL
Park et al., 2021 Aim: To evaluate the clinical results of metronidazole administration in combination with minocycline as a local adjunct for the non-surgical treatment of peri-implantitis (Park et al., 2021)	Multicenter study 118 subjects; three groups	Group a: mechanical debridement and metronidazole and minocycline ointment Group b: mechanical debridement and minocycline ointment Group c: mechanical debridement only	12 weeks	The additive use of metronidazole or minocycline results in significantly higher treatment success rates compared with mechanical debridement alone in the non-surgical treatment of peri-implantitis	PPD, PS, SoP, BL
Shibli et al., 2019 Aim: To evaluate the effects of adjunctive systemic antibiotic therapy with metronidazole and amoxicillin in patients undergoing non-surgical subgingival debridement for peri-implantitis (Shibli et al., 2019)	40 subjects; two groups (20 experimental and 20 control)	Patients in the experimental group were treated with non-surgical peri-implant debridement and adjuvant systemic antibiotic (metronidazole and amoxicillin) for 14 days. Patients in the control group received non-surgical peri-implant debridement and a placebo	14 days and 3, 6, and 12 months	The results of this study do not support the adjunctive use of systemic metronidazole and amoxicillin in the non-surgical treatment of peri-implantitis. Current non-surgical treatment protocols are often insufficient to treat severe cases of peri-implantitis	PPD, CAL, BoP, PS, SoP, VBL

PPD, probing pocket depth; REC, recession; CAL, clinical attachment level; BoP, bleeding on probing; PS, plaque score; BS, bleeding score; PIPD, peri-implant pocket depth; SoP, suppuration on probing; BL, bone level; RBL, radiographic bone loss; VBL, vertical bone loss.

**Table 4** describes the specific characteristics of the studies. Three indicated the pathogen studied (De Waal et al., 2021; Park et al., 2021; Blanco et al., 2022), two the type of implant (De Waal et al., 2021; Polymeri et al., 2022), and two more (Park et al., 2021; Blanco et al., 2022) used C-reactive protein (CRP) to detect and quantify bacterial DNA. To assess peri-implant marginal radiographic bone levels, all the included studies used intraoral radiographs. The Shapiro–Wilk test was the most commonly used in statistical analyses (Park et al., 2021; Blanco et al., 2022; Polymeri et al., 2022). A history of associated periodontitis was only considered in three of the studies (De Waal et al., 2021; Blanco et al., 2022; Polymeri et al., 2022).

**Table 4:** Specific characteristics of the studies included.

Study	Type of implant	Implant location	Radiographic test	Non-surgical treatment	Pathogens studied; method	Statistical method	Demographic data
Blanco et al. (2022)	Not reported	Not reported	Periapical radiograph	Non-surgical debridement using ultrasonic stainless steel scaling inserts. Chlorhexidine 0.12%	*Aggregatibacter actinomycetemcomitans*, *Campylobacter rectus*, *Fusobacterium nucleatum*, *Porphyromonas gingivalis*, *Tannerella forsythia* qCRP	Shapiro–Wilk test	Age, sex, smoking status, history of periodontitis, systemic diseases
Polymeri et al. (2022)	Nobel, Straumann, Biomet 3i, Other	Maxilla/mandible (anterior/posterior)	Periapical radiograph	Ultrasonic devices with polyether ether ketone fiber tips and carbon fiber-reinforced plastic handheld instruments. Chlorhexidine 0.12%	Not reported	Cohen’s *d*; Shapiro–Wilk test	Age, sex, BMI, smoking status, history of periodontitis, full-mouth plaque score, dental status (partially edentulous/fully edentulous), number of dental implants
De Waal et al. (2021)	Alpha-Bio Tec, Camlog, Dentium, Dentsply Sirona, MIS, Neobiotech, Nobel, Biocare, Straumann, Zimmer, Biomet	Maxilla/mandible	Periapical radiograph	Air polisher with subgingival tip. Ultrasonic instruments. Chlorhexidine 1%	*Aggregatibacter actinomycetemcomitans*, *Porphyromonas gingivalis*, *Prevotella intermedia*, *Tannerella forsythia*, *Parvimonas micra*, *Fusobacterium nucleatum*, *Treponema denticola* Method not reported	Cohen’s *d*; Mann–Whitney *U* test	Sex, smoking status, history of periodontitis, alcohol consumption, diabetes, plaque index
Park et al. (2021)	Implant surface non-modified/modified	Maxilla/mandible (anterior/posterior)	Periapical radiograph	Not reported	*Porphyromonas gingivalis*, *Tannerella forsythia*, *Treponema denticola*, *Fusobacterium nucleatum*, *Prevotella intermedia*, *Prevotella nigrescens*, *Peptostreptococcus micros*, *Eubacterium nodatum*, *Campylobacter rectus*, *Eikenella corrodens* CRP	Shapiro–Wilk test	Age, sex
Shibli et al. (2019)	Not reported	Not reported	Periapical radiograph	Not reported	39 bacterial species according to the microbial complexes described by Socransky et al. (1998)	Wilcoxon test	Not reported

qCRP, quantitative C-reactive protein; BMI, body mass index.

### 3.2 Methodological quality

All the studies included in the meta-analysis achieved a score on the Jadad scale compatible with high methodological quality (≥3 points), with the study by Blanco et al. (2022) achieving the highest score (**Table 5**).

**Table 5:** Jadad quality score of randomized clinical trials (RCTs) included in the meta-analysis.

Study	Randomization	Blinding	Dropouts	Total score
Blanco et al., 2022 (Blanco et al., 2022)	4	5	5	14
Polymeri et al., 2022 (Polymeri et al., 2022)	3	2	5	10
De Waal et al., 2021 (De Waal et al., 2021)	4	2	5	11
Park et al., 2021 (Park et al., 2021)	3	4	1	8
Shibli et al., 2019 (Shibli et al., 2019)	4	4	1	9

Each study was assigned a score of 0–5. Mode value: 10.4 ± 2.30.

### 3.3 Risk of bias assessment

Of the five included studies (Shibli et al., 2019; De Waal et al., 2021; Park et al., 2021; Blanco et al., 2022; Polymeri et al., 2022), only four were included in the meta-analysis (Shibli et al., 2019; De Waal et al., 2021; Park et al., 2021; Blanco et al., 2022); the study by Polymeri et al. (2022) was excluded for presenting data in a non-analyzable form.

**Figure 3:**
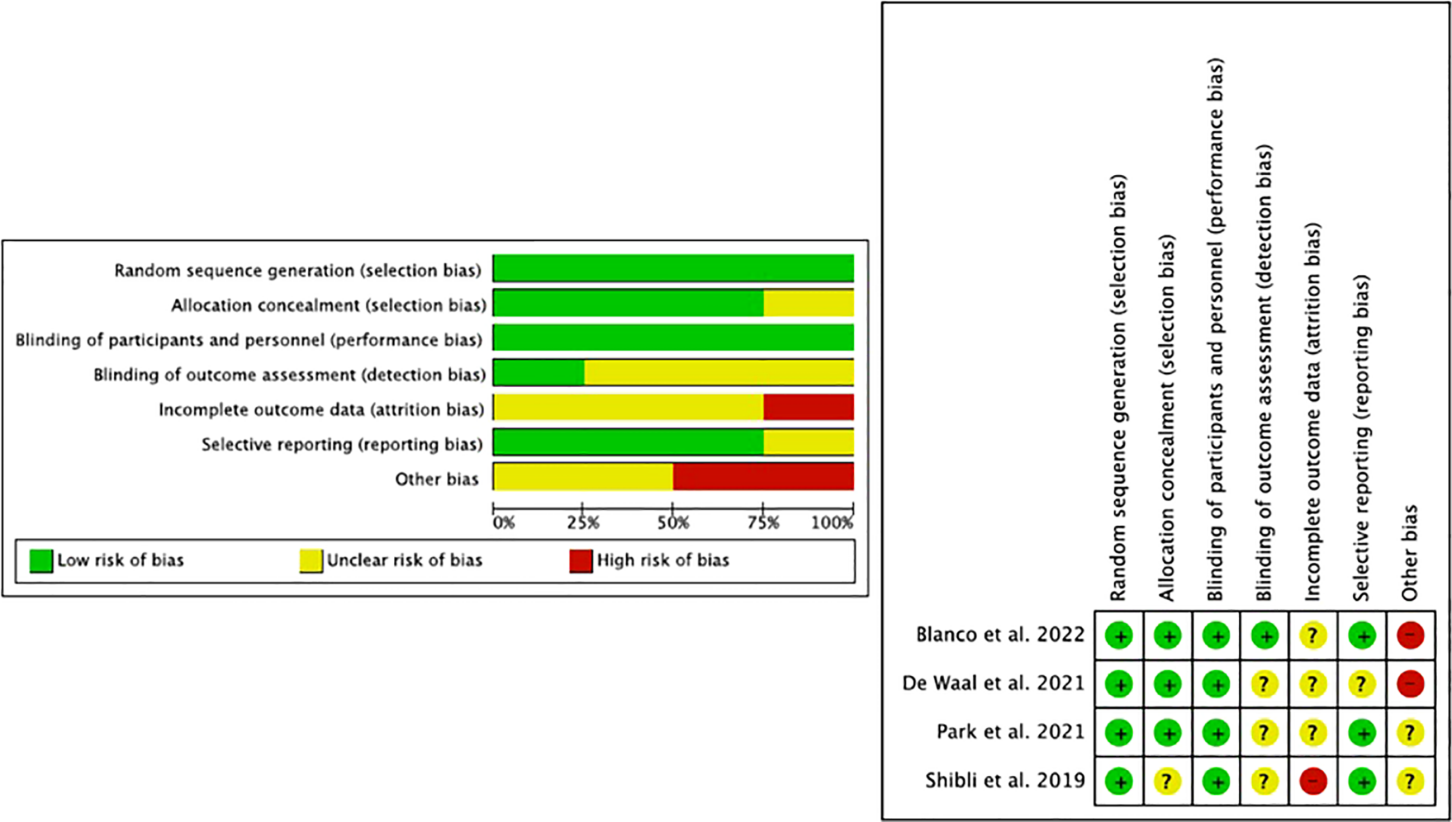
Risk of bias assessment.

All studies met the domains of random sequence generation and blinding of participants and personnel; one study was biased in the incomplete outcome domain (Shibli et al., 2019), and all had unclear data in the domains of the incomplete outcome, blinding of outcome assessment, and allocation concealment. The study by Blanco et al. (2022) was the highest rated (**Figure 3**).

### 3.4 Qualitative synthesis

The selected studies included a total of 289 patients, and follow-up periods ranged from 2 weeks to 1 year. The studies with the most complete follow-up were those by Blanco et al. (2022) and Shibli et al. (2019) at 3, 6, and 12 months. The plaque score (PS) parameter was considered in all the included studies, followed by the probing pocket depth (PPD) (Shibli et al., 2019; De Waal et al., 2021; Park et al., 2021; Blanco et al., 2022) and suppuration on probing (SoP) (Shibli et al., 2019; De Waal et al., 2021; Park et al., 2021; Polymeri et al., 2022) parameters. The bleeding score (BS) parameter was measured in only one study (De Waal et al., 2021) (**Table 3**). All studies included in the meta-analysis (Shibli et al., 2019; De Waal et al., 2021; Park et al., 2021; Blanco et al., 2022) used radiographic parameters to assess peri-implant marginal bone levels (**Table 4**). The bone level (BL) parameter was measured in two studies (De Waal et al., 2021; Park et al., 2021), radiographic bone loss (RBL) in one (Blanco et al., 2022), and vertical bone loss (VBL) in another (Shibli et al., 2019).

### 3.5 Quantitative synthesis and meta-analysis results

Only four of the included studies were used for meta-analysis (Shibli et al., 2019; De Waal et al., 2021; Park et al., 2021; Blanco et al., 2022). The highest weight was given to the multicenter study by Park et al. (2021) due to the high number of patients included in the study. Meta-analysis of adverse outcomes could not be performed due to a lack of data.

**Figure 4:**
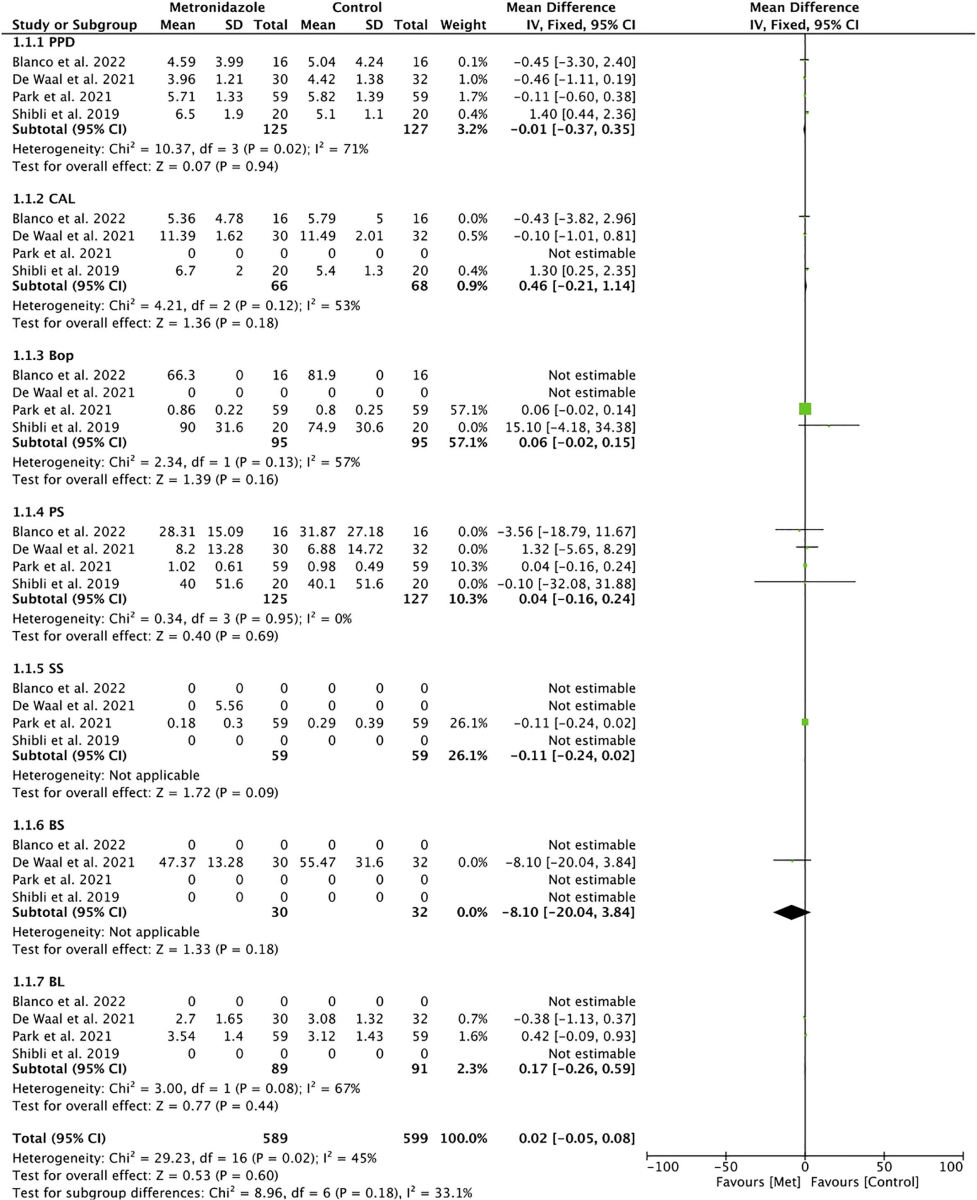
Forest plot for the grouped studies.

**Figure 5:**
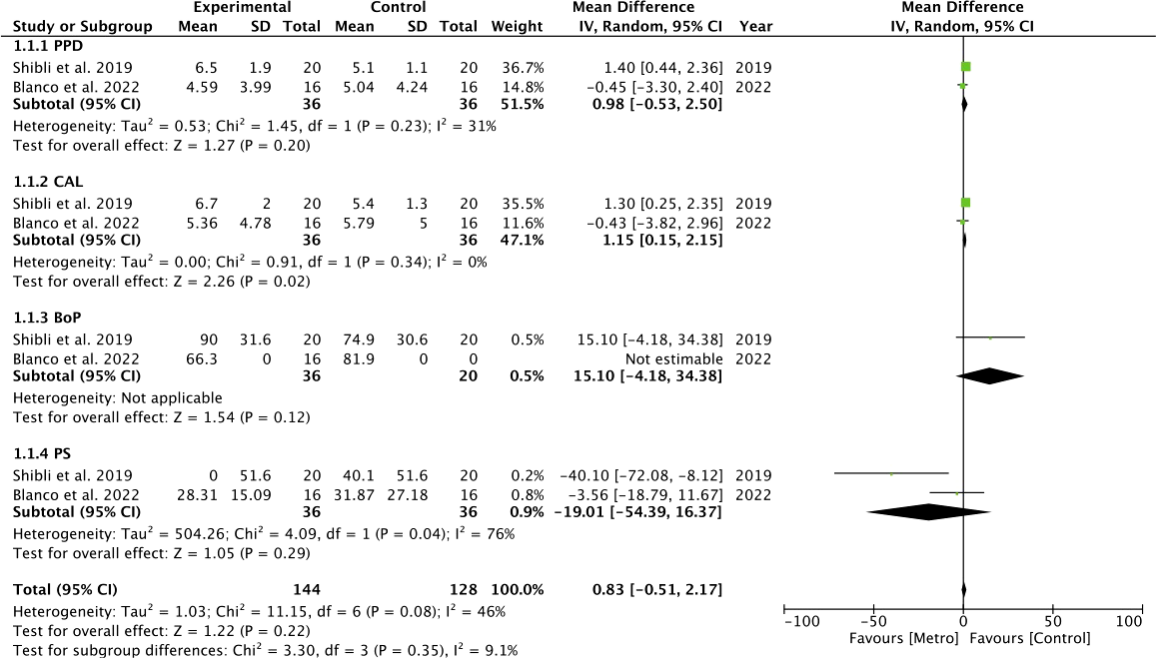
Forest plot for subgroup 1 (PPD, CAL, BoP, PS).

**Figure 6:**
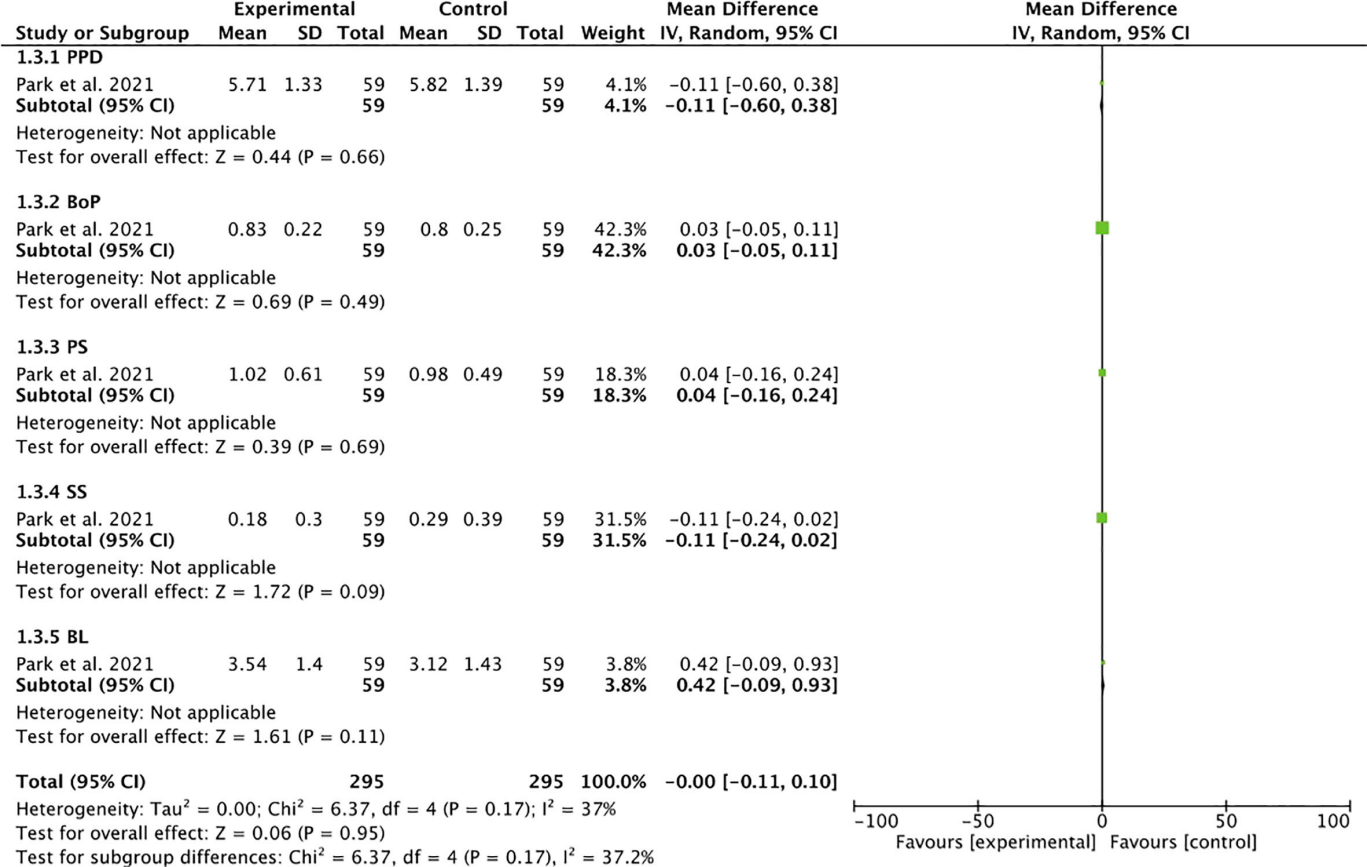
Forest plot for subgroup 3 (PPD, BoP, PS, SS, BL).

Heterogeneity was moderate in the analysis of grouped studies (*I* ^2 ^= 45%) (**Figure 4**). In the analysis by subgroups, total homogeneity was found in subgroup 2 (*I* ^2 ^= 0%) (**Figure 1**); subgroups 1 and 3 obtained moderate heterogeneity (*I* ^2 ^= 46% and *I* ^2 ^= 37%, respectively) (**Figures 5**, **6**). Statistical significance was found only in the pooled analysis of the studies (*p* = 0.02) (95% CI) (**Figure 4**). Subgroup 2, which analyzed studies investigating PPD, clinical attachment level (CAL), PS, suppuration score (SS), BS, and BL (**Figure 1**), was the furthest from statistical significance (*p* = 0.69).

**Figure 7:**
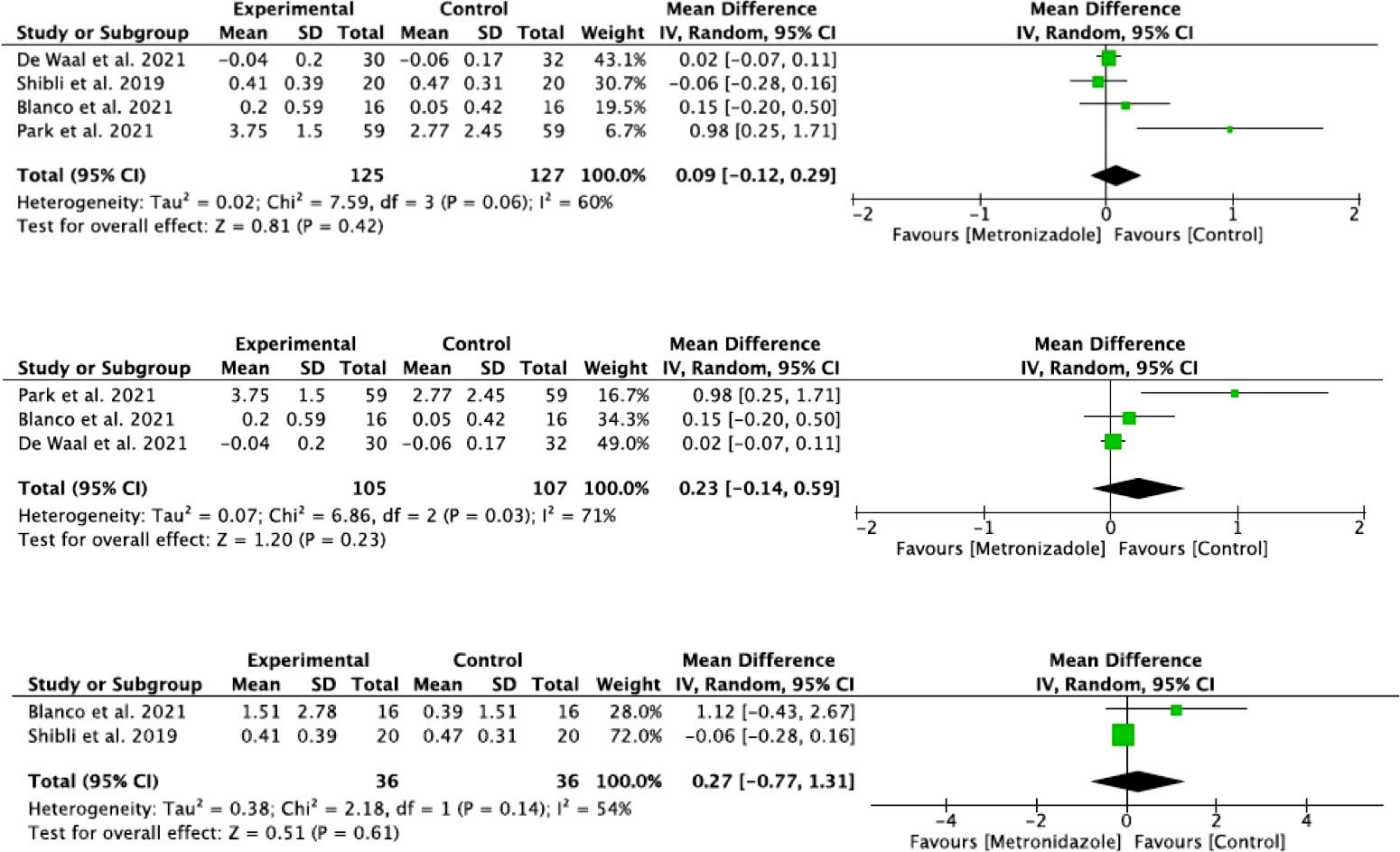
Forest plot for radiographic evaluation of all studies: radiographic evaluation of studies with a 3-month follow-up and radiographic evaluation of studies with a 12-month follow-up.

**Figure 8:**
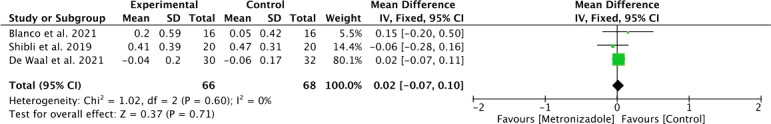
Forest plot for BL of studies in which systemic antibiotics were used.

Three subgroups were performed for the radiographic values reported on peri-implant marginal bone levels: the first from the pooled studies included in the meta-analysis (Shibli et al., 2019; De Waal et al., 2021; Park et al., 2021; Blanco et al., 2022), the second from studies with a 3-month follow-up (De Waal et al., 2021; Park et al., 2021; Blanco et al., 2022), and the third from studies with a 12-month follow-up (Shibli et al., 2019; Blanco et al., 2022) (**Figure 7**). Heterogeneity was moderate for the three subgroups (*I* ^2^ < 75%). Statistical significance (*p* = 0.03) was found only in the 3-month follow-up subgroup. The bone level loss was also analyzed separately in studies using systemic antibiotics (Shibli et al., 2019; De Waal et al., 2021; Blanco et al., 2022), finding overall homogeneity (*I* ^2 = ^0%), so a fixed-effects model was performed; however, no statistical significance was found between groups (*p* = 0.60) (**Figure 8**). The study by Park et al. (2021), in which topical antibiotics were used, could not be analyzed because it only provided data at the bone level at the baseline of treatment.

### 3.6 Publication bias

Although we are aware that publication bias is not advisable when the meta-analysis is composed of fewer than 10 studies (Higgins and Thompson, 2002) (as is our case), we preferred to include it in the overall analysis.

**Figure 9:**
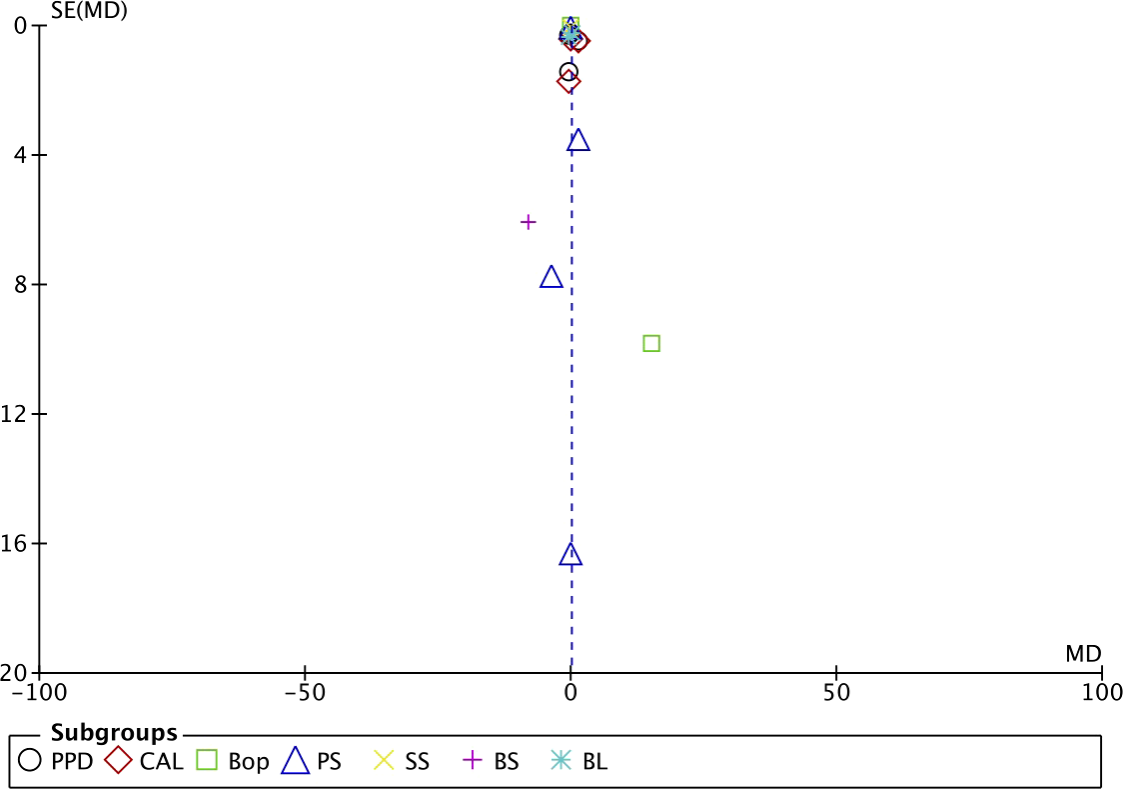
Funnel plot.

The funnel plot analysis suggested moderate publication bias. In general, the estimated effect is associated with the horizontal axis and the sample size with the vertical axis. The studies that measured BoP (Shibli et al., 2019; Blanco et al., 2022; Polymeri et al., 2022) showed the greatest asymmetry (**Figure 9**).

## 4 Discussion

Systematic reviews on the efficacy of adjuvant antibiotics in the treatment of peri-implantitis are scarce, and we did not find any meta-analysis that analyzed the efficacy of this antimicrobial (i.e., metronidazole), so this systematic review aimed to evaluate the efficacy of metronidazole as an adjuvant in the treatment of peri-implantitis. In total, five RCTs were included in the analysis, of which four were analyzed and one was eliminated for providing non-analyzable data.

The absence of BoP is evidence of the absence of inflammation, which should be the first target in the treatment of peri-implantitis, and according to recent recommendations, the results of peri-implantitis treatment should be evaluated after 6 months of healing, based on outcomes including multiple parameters: bone fill, peri-implant soft tissue recession, BoP, SoP, and PPD (Sanz and Chapple, 2012). However, in our meta-analysis, only the studies by Blanco et al. and Shibli et al (Shibli et al., 2019; Blanco et al., 2022). performed a 3-, 6-, and 12-month follow-up; the studies by De Waal et al. and Park et al (De Waal et al., 2021; Park et al., 2021). performed a single 12-week follow-up.

Mombelli and Decaillet (Mombelli and Décaillet, 2011) highlighted the growth of gram-negative anaerobic species in peri-implant pockets, compatible with peri-implantitis, and that peri-implant disease can be considered a mixed anaerobic infection, warning of the benefits of combined mechanical and chemical treatments. All the studies included in our meta-analysis combined mechanical debridement and adjuvant antibiotic treatment; however, in this aspect, several studies have demonstrated the benefits of the use of systemic antibiotics as an adjunct to non-surgical treatment of peri-implantitis (Liñares et al., 2019; Nart et al., 2020), although a Cochrane systematic review (Esposito et al., 2012) warned that there is no reliable evidence to suggest which interventions might be the most effective in the treatment of this pathology. A recent systematic review and meta-analysis by Ramanauskaite et al. (2021) found that adjunctive reconstructive measures in conjunction with surgical treatment of peri-implantitis were beneficial in terms of radiographic reduction of the bony defect and reduced soft tissue recession, despite not improving mucosal inflammation; likewise, systemic antibiotics did not provide any benefit to the outcomes of non-reconstructive surgical treatment of peri-implantitis.

According to the results obtained in our study, the pooled RCT analysis demonstrated the effect of metronidazole as an adjuvant treatment after 12 weeks (*p* = 0.02). A statistically significant result (*p* = 0.03) was also obtained in the analysis of studies with shorter follow-up (3 months).

Blanco et al. (2022) obtained at 12 months a significant reduction in PPD (2.53 *vs*. 1.02 mm) and CAL (2.14 *vs*. 0.53 mm) in the test group compared with the control group. Similarly, Shibili et al (Shibli et al., 2019). with metronidazole plus adjuvant amoxicillin found at 12 months a probing reduction of 3.1 mm in the antibiotic group *vs*. 1.8 mm in the control group, despite including in their study compromised cases, with PD >5 mm, peri-implant bone loss >4 mm, and BoP and/or SoP. Studies, such as Cionca et al. using systemic metronidazole and amoxicillin, significantly improved clinical outcomes at 6 months after non-surgical full-mouth periodontal debridement, thus significantly reducing the need for additional treatment (Cionca et al., 2009). In contrast, the studies by Polymeri et al., de Waal et al., and Park et al (De Waal et al., 2021; Park et al., 2021; Polymeri et al., 2022). found no clinical benefits with adjunctive antibiotic treatment; the results coincided with Stein et al. (2017) who found that, in a study of 45 patients with chronic periodontitis, carriers of 164 implants with peri-implantitis, treatment with adjunctive metronidazole/amoxicillin had no significant benefits in the changes in mean PPD, CAL, and BoP values at 12 months of treatment. Blanco et al. (2022), with the use of metronidazole as adjuvant treatment, found a significantly higher radiographic bone gain at 12 months in the test group compared with the control group (2.15 mm test *vs*. 0.95 mm control). In contrast, Shibli et al., with metronidazole and amoxicillin and the same follow-up time, found no significant differences between the groups (Shibli et al., 2019). In this regard, some authors have even suggested that the prescription of amoxicillin plus metronidazole as an adjunct to non-surgical treatment should be limited to patients with specific microbiological profiles, especially those positive for *A. actinomycetemcomitans*, as this germ is known to resist mechanical treatment particularly well (Mombelli et al., 2000; Dannewitz et al., 2007); however, Mombelli et al. (2013), in a longitudinal study of 82 patients with 41 positive cases for *A. actinomycetemcomitans* and 41 negative cases, also found no specific benefit from the use of metronidazole in combination with amoxicillin.

Certain similarities in terms of biomarkers of bone destruction and causative microbial agents, in peri-implant and periodontal pathologies and the frequent coincidence of both in the same patient, have led some researchers to investigate both pathologies together or to design studies on peri-implantitis based on those performed on periodontitis (Mombelli and Décaillet, 2011), although Liu et al. and Zhang et al (Liu et al., 2020; Zhang et al., 2022). observed that peri-implantitis and periodontitis have significantly different microRNA and long-chain RNA expression profiles, indicating that osteoclast differentiation pathways are more active in peri-implantitis than in periodontitis.

On the other hand, it is known that an individual’s immune response to bacterial aggression is influenced by genetic and epigenetic factors, as well as environmental factors. It has been shown that inflammatory diseases, such as peri-implantitis and periodontitis, can be enhanced by epigenetic modifications in certain subjects (Larsson et al., 2022). A recent systematic review concluded with the need for future research to explore the functional role of specific microRNAs and their possible role as therapeutic targets (Asa'ad et al., 2020). A correlation between IL-1-specific gene polymorphisms and peri-implant bone loss has also been described in smoking subjects, although smoking is not considered a conclusive risk factor (Feloutzis et al., 2003; Dreyer et al., 2018; Schwarz et al., 2018).

The present meta-analysis has some limits that must be emphasized. On the one hand, the included studies used local and systemic adjuvant antibiotics; on the other hand, only the study by Blanco et al. (2022) used metronidazole as an adjuvant treatment; all the others (Shibli et al., 2019; De Waal et al., 2021; Park et al., 2021) used metronidazole combined with other antibiotics. Another aspect to consider would be the different implant surfaces used in the studies. It has been reported that the mean peri-implant bone loss around moderately and minimally roughened surfaces is less than around roughened surfaces; however, certain studies have reported that the clinical impact of surface roughness alone on bone loss and the risk of peri-implantitis appears to be rather limited and of minimal clinical importance (Renvert et al., 2011; De Bruyn et al., 2017). Only three of the studies included in our meta-analysis took into account implant type or surface modification (De Waal et al., 2021; Park et al., 2021; Polymeri et al., 2022), yet all considered age, sex of patients, and location of inserted implants, although Dreyer et al (Feloutzis et al., 2003), in a systematic review, reported with a medium–high level of evidence that the age and sex of the patients were not related to peri-implantitis.

On the other hand, Song et al. (2020) proposed that implants placed in anterior regions, both maxillary and mandibular, would have a higher prevalence of peri-implantitis compared with posterior regions, and in our meta-analysis, only two of the included studies (De Waal et al., 2021; Park et al., 2021) reported on the site of implant placement; they also combined implants in the anterior and posterior sites. However, some studies have drawn attention to the “clustering effect,” considering that implants placed in the same mouth should not be considered as independent (Pikner and Gröndahl, 2009).

The outcome reports of the included studies were conducted at different time periods, and although Sanz et al (Sanz and Chapple, 2012). recommend evaluating the results of peri-implantitis treatment 6 months after healing, we have not found unanimity in this recommendation; a systematic review by Heitz-Mayfield and Mombelli chose a 12-month re-evaluation period to evaluate the satisfactory outcome of treatment (Heitz-Mayfield and Mombelli, 2014).

Finally, although all studies used radiographic evaluation by intraoral radiography to assess the peri-implant bone loss, there are other useful radiographic techniques, such as multislice computed tomography and cone-beam volumetric imaging, which offer certain advantages to implant dentistry, such as the representation of intraosseous lesions in three planes, on a real scale and without overlapping or distortion. In addition, computer-assisted image analysis, such as subtraction radiography, allows the detection of small changes in bone density (Heitz-Mayfield, 2008; Naveau et al., 2019; Trivedi et al., 2022). The different cutoff levels reported for the different clinical parameters and the different methods of statistical analysis in the included studies should also be taken into account; only three of them (Park et al., 2021; Blanco et al., 2022; Polymeri et al., 2022) resorted to the Shapiro–Wilk test to contrast the normality of the data.

All this could bias the results, and therefore, the results obtained in our meta-analysis should be taken with caution.

Therefore, we consider that the decision on a specific adjuvant antibiotic treatment should be made individually for each patient, taking into account the severity and extent of peri-implantitis and possible concomitant periodontitis, as well as the number of affected areas requiring treatment. Likewise, the potential risk of the development of strains resistant to metronidazole or to the chosen antimicrobial combination should be taken into account, and the protocol to be followed in each indicated situation should be carefully decided (Ardila et al., 2010; Veloo et al., 2012). On the other hand, the approach to incipient peri-implantitis is of vital importance, since bone defects in advanced stages require complete debridement and repositioning of the marginal mucosa to allow the patient effective oral hygiene, generally compromising the esthetic outcome of prosthetic restorations (Figuero et al., 2014).

## 5 Conclusions

Adjuvant local or systemic administration of metronidazole in the treatment of peri-implantitis remains of questionable efficacy and with serious discrepancies among investigators.

Due to the heterogeneity of the types of treatment, the reported administration protocols, and, ultimately, the heterogeneity of the studies, we have not been able to draw definitive conclusions about its effect in the adjuvant treatment of peri-implantitis.

In certain pathologic situations of peri-implantitis, metronidazole, alone or in combination with other antibiotics, could be beneficial as an adjuvant to surgical treatment, always weighing the benefits against the disadvantages.

Long-term RCTs with standardized methodologies would be desirable and justifiable to determine the exact role of metronidazole as an adjuvant treatment for peri-implantitis.

## Author contributions

Conceptualization: NL-V and AL-V. Methodology: NL-V. Validation: JB-R. Formal analysis: NL-V. Resources: AL-V. Data curation: NL-V. Writing—original draft preparation: NL-V. Writing—review and editing: NL-V and AL-V. All authors contributed to the article and approved the submitted version.
